# An IRF5 Decoy Peptide Reduces Myocardial Inflammation and Fibrosis and Improves Endothelial Cell Function in Tight-Skin Mice

**DOI:** 10.1371/journal.pone.0151999

**Published:** 2016-04-06

**Authors:** Dorothee Weihrauch, John G. Krolikowski, Deron W. Jones, Tahniyath Zaman, Omoshalewa Bamkole, Janine Struve, Savin Pillai, Paul S. Pagel, Nicole L. Lohr, Kirkwood A. Pritchard

**Affiliations:** 1 Departments of Anesthesiology, Medical College of Wisconsin, Milwaukee, Wisconsin, United States of America; 2 Department of Surgery, Division of Pediatric Surgery, Medical College of Wisconsin, Milwaukee, Wisconsin, United States of America; 3 Children’s Research Institute, Medical College of Wisconsin, Milwaukee, Wisconsin, United States of America; 4 Orthopedic Surgery, Medical College of Wisconsin, Milwaukee, Wisconsin, United States of America; 5 Clement J. Zablocki Veterans Affairs Medical Center, Milwaukee, Wisconsin, United States of America; 6 Department of Medicine, Division of Cardiology, Medical College of Wisconsin, Milwaukee, Wisconsin, United States of America; Medical University of South Carolina, UNITED STATES

## Abstract

Interferon regulatory factor 5 (IRF5) has been called a “master switch” for its ability to determine whether cells mount proinflammatory or anti-inflammatory responses. Accordingly, IRF5 should be an attractive target for therapeutic drug development. Here we report on the development of a novel decoy peptide inhibitor of IRF5 that decreases myocardial inflammation and improves vascular endothelial cell (EC) function in tight-skin (Tsk/+) mice. Biolayer interferometry studies showed the Kd of IRF5D for recombinant IRF5 to be 3.72 ± 0.74x10^-6^M. Increasing concentrations of IRF5D (0–100 μg/mL, 24h) had no significant effect on EC proliferation or apoptosis. Treatment of Tsk/+ mice with IRF5D (1mg/kg/d subcutaneously, 21d) reduced IRF5 and ICAM-1 expression and monocyte/macrophage and neutrophil counts in Tsk/+ hearts compared to expression in hearts from PBS-treated Tsk/+ mice (p<0.05). EC-dependent vasodilatation of *facialis* arteries isolated from PBS-treated Tsk/+ mice was reduced (~15%). IRF5D treatments (1mg/kg/d, 21d) improved vasodilatation in arteries isolated from Tsk/+ mice nearly 3-fold (~45%, p<0.05), representing nearly 83% of the vasodilatation in arteries isolated from C57Bl/6J mice (~55%). IRF5D (50μg/mL, 24h) reduced nuclear translocation of IRF5 in myocytes cultured on both Tsk/+ cardiac matrix and C57Bl/6J cardiac matrix (p<0.05). These data suggest that IRF5 plays a causal role in inflammation, fibrosis and impaired vascular EC function in Tsk/+ mice and that treatment with IRF5D effectively counters IRF5-dependent mechanisms of inflammation and fibrosis in the myocardium in these mice.

## Introduction

IRF5 is a member of the interferon regulatory factor (IRF) family, a group of transcription factors with diverse roles, including virus-mediated activation of interferon and regulation of cell growth, differentiation, and apoptosis and modulation of immune system activity. In recent years it has been reported that IRF5 also controls the balance between type-1 and type-2 immune responses. Because type-1 responses promote inflammation and destruction of pathogens and type-2 responses promote tissue repair and growth, the ability of IRF5 to mediate the balance between these pathways earned it the reputation of being a “master switch” in immunology. Notably, chronic IRF5 activation enhances apoptosis, a characteristic feature of cancer [1] as well as autoimmune disorders such as inflammatory bowel disease, lupus erythematosus and scleroderma [2–4]. Although substantial evidence exists linking IRF5 to autoimmune disease and a number of reports suggest IRF5 may be an important therapeutic target for treating autoimmune disease [5–7], inhibitors for IRF5 remain lacking.

Scleroderma or systemic sclerosis (SSc) is a group of autoimmune fibrotic disorders that affect approximately 150,000 patients in the United States [8]. Marked increases in fibrosis of the skin and internal organs concomitant with enhanced apoptosis, characterize this dreadful disease. One of the clinical features of SSc is a marked increase in myocardial inflammation, fibrosis and heart failure [9–13]. Interestingly, tight skin (Tsk/+) mice, a murine model of autoimmunity, inflammation and fibrosis that has been used to study mechanisms of SSc [12,14], also develop myocardial inflammation and fibrosis and heart failure in ways that mimic heart disease in humans with SSc [15–17].

Previously, we reported that 4F, an apoA-I mimetic, reduced myocardial inflammation and fibrosis and heart failure in Tsk/+ mice by a mechanism that appeared to be mediated in part, by the ability of 4F to bind IRF5 [12]. As 4F possesses many mechanisms of action, its ability to prevent myocardial inflammation and heart failure in Tsk/+ mice cannot be attributed exclusively to its ability to inhibit IRF5. Accordingly, we developed a decoy peptide inhibitor of IRF5 to investigate the role of IRF5 in myocardial and endothelial dysfunction in Tsk/+ mice. Decoy peptides such as IRF5D are straight forward to design. This is especially true if the 3D structure of the protein is known, as was the case with IRF5 [18,19]. Even without the 3D structure, small peptides that bind to and block proteins can be identified. Blocking peptides can be identified using phage display or via analysis of binding domains of known binding partners. Alternatively, but more costly, is a technique that we call “peptide walking.” Essentially, this technique uses a series of overlapping peptides spanning an entire protein or more conservatively, a portion of a protein that contains a putative binding domain. The peptides are added individually to aliquots of cell homogenates and interactions determined using pull-down assays and immunoblots. The peptide that is most effective at disrupting protein interactions contains the sequence of a potential inhibitor. We used this approach to develop SB2, a small peptide derived from eNOS that could disrupt hsp90-eNOS interactions and impair vascular EC function [20]. The more one knows about the binding partners of a target protein, the more opportunities they have for designing blocking peptides.

In the present report we show that targeting IRF5 with a blocking peptide decreases myocardial inflammation and fibrosis and improves vascular endothelial function in Tsk/+ mice.

## Methods

This study was carried out in strict accordance with the recommendations in the Guide for the Care and Use of Laboratory Animals of the National Institutes of Health. The protocol was approved by the Institutional Animal Care and Use Committee (Protocol: AUA#1517). All research involving mice was conducted in conformity with PHS policy.

### Biolayer Interferometry (BLI)

Protein-protein interactions were determined by BLI on an Octet RED (forteBio, Inc, Menlo Park, CA). Recombinant IRF5 was made and purified by Top Gene Technologies (Montreal, Quebec, Canada) and biotin labeled by our laboratories using an EZ-Link NHS-PEG4-Biotin kit (cat no. 21329, Thermo Fisher, Rockford, IL) at a ratio of 1:1, the optimal ratio for binding and packaging target proteins onto streptavidin (SA) biosensors (MR18-0009, forteBio, Menlo Park, CA). Biotin-labeled IRF5 (B-IRF5) was loaded onto SA-biosensors in phosphate buffered (PBS) for 15 minutes to ensure saturation. The B-IRF5-loaded SA biosensors were then incubated in PBS containing increasing concentrations of IRF5D for 10 minutes and rates of association and dissociation determined as described [12,18,21].

### Effects of IRF5D on EC Proliferation and Apoptosis

Effects of IRF5D on EC proliferation and apoptosis were determined by measuring changes in caspase activity, an index of apoptosis, and bioreduction of the tetrazolium dye, an index of proliferation. Briefly human umbilical vein endothelial cells (EC) were cultured in 96 well plates coated with C57Bl/6J cardiac matrix and then treated with increasing concentrations of IRF5D (0–100 μg/mL). After 72 hours, effects of IRF5D on EC proliferation were determined using the CellTiter AQueous One Solution Cell Proliferation Assay kit (Promega, Madison, WI). Changes in absorbance were measured on a Bio-Tek microplate reader. Effects of IRF5D on EC apoptosis were determined by adding the green detection reagent, CellEvent^TM^ Caspase-3/7 Substrate, (Life technologies, Grand Island, NY) was added and fluorescence measured on a Bio-Tek microplate reader at the same time point. The experiments were run in triplicates.

### Effects of IRF5D Treatments on Myocardial IRF5 and ICAM Expression

Tsk/+ and C57Bl/6J mice were treated with either PBS or IRF5D (1mg/kg/d, subcutaneous) for 21 days. Hearts were isolated and homogenates prepared as previously described [12]. Western blot analysis of homogenate proteins was performed using polyclonal anti-IRF5 and anti-ICAM-1 antibodies diluted 1:1000 (Santa Cruz Biotechnologies, Santa Cruz, CA) as primary antibodies. Bands of identity in the blots were visualized using ECL plus reagent (GE Healthcare, Piscataway, NJ), horseradish peroxidase (HRP) -conjugated donkey anti-mouse IgG and HRP-anti-goat IgG (1:10,000 dilution, Santa Cruz Biotechnologies, Santa Cruz, CA) and X-ray film to detect ICAM-1 and IRF5, respectively. Densitometry of bands in the film was measured using NIH Image J (Bethesda, MD) software and densities normalized to control bands. Western blots were performed in triplicate using hearts isolated from 4 mice in each test group [12].

### Effects of IRF5D Treatment on the Number of Monocytes/Macrophages and Neutrophils

Tsk/+ mice were treated with PBS and IRF5D as described above. Hearts were snap-frozen and frozen sections (10 μm) were cut. These sections were fixed in 1% paraformaldehyde (Sigma-Aldrich, St. Louis, MO) in phosphate buffered saline for 20 minutes at room temperature and permeabilized for 5 minutes in 0.5% Triton–X 100 in phosphate buffered saline (Sigma-Aldrich, St. Louis, MO). Primary monocyte/macrophage marker anti-rabbit CD64 and primary neutrophil marker antibody anti-rat NIMP (Santa Cruz Biotechnologies, Santa Cruz, CA) were incubated for 30 minutes at 37°C. This was followed by 3 wash steps with phosphate buffered saline at room temperature. The secondary antibodies (anti-rabbit Alexa 488 conjugated and anti-rat Alexa 488 conjugated, Life Technologies, Grand Island, NY) were applied accordingly for 30 minutes at 37°C. The nuclei were counterstained with DAPI (Life Technologies, Grand Island, NY). CD64 and NIMP fluorescence was visualized using confocal microscopy (Nikon Eclipse TE 200-U microscope with EZ C1 laser scanning software, Melville NY) using excitation wavelengths 488 and emission wavelengths of greater than 530 nm for CD64 and NIMP. DAPI was excited with 360nm and emitted at 460nm in blue. The number of CD64 and NIMP positive cells were counted (n = 10 images per antibody, from 3 different mice in each group). The data are expressed as number of counted cells.

### Effects of IRF5D on EC-dependent Vasodilatation

Acetylcholine (ACh)–induced, EC-dependent vasodilatation was determined on isolated and pressurized *facialis* arteries as previously described [21]. Briefly, isolated *facialis* arteries were cannulated and pressurized. Changes in vessel diameters were determined by video microscopy. Vessels were stimulated by adding sequentially increasing concentrations of ACh (10^−7^ to 10^−4^ M) and changes in vessel diameter recorded 2 minutes later [22].

### Isolation of Cardiac Extracellular Matrix

Hearts were isolated from C57Bl/6J and Tsk/+ mice treated with or without IRF5D as described above. Hearts were finely minced and pieces incubated and agitated in 1% sodium dodecyl sulfate (SDS) for 18 hours at room temperature to dissolve the cells. The next day, a Triton X-100 wash (30 minutes with agitation) was performed, followed by a thorough PBS rinse (15 minutes with agitation). The isolated cardiac extracellular matrix was then snap-frozen in liquid nitrogen and pulverized using a mortar and pestle that was pre-cooled in liquid nitrogen. The powdered matrix was suspended in Dulbecco’s PBS and sonicated on ice for 30 seconds. Protein content was determined using Bradford Reagent (Life Technologies, Grand Island, NY).

### IRF5 Nuclear Translocation

Cell culture plates and slides were coated with the cardiac extracellular matrices (20 μg/ml, final concentration) described above. Myocyte cultures were treated with or without IRF5D (50 μg/mL, 24 h). IRF5 translocation from the cytosol to the nucleus was determined on cultured myocytes using immunofluorescence confocal microscopy. Anti-IRF5 antibody (Santa Cruz Biotechnologies) was used as the primary antibody and Alexa 488-labeled goat anti-mouse IgG antibody (Molecular Probes) was used as the secondary antibody and DAPI as a nuclear stain. Fluorescent images were captured with a fluorescent Nikon Eclipse microscope and fluorescence intensity in the cytosol and nuclei of the cells in the captured images was measured using NIKON Element imaging software as previously described [12].

### Statistical Analysis

Statistical analysis of data within and between groups was performed with analysis of variance (ANOVA) for repeated measures followed by Bonferroni’s modification of Student’s t-test. The null hypothesis was rejected when p = 0.05. All data are expressed as mean standard error of the mean (SEM) except were specifically stated as being SD.

## Results

### Design of IRF5D

**Fig 1:**
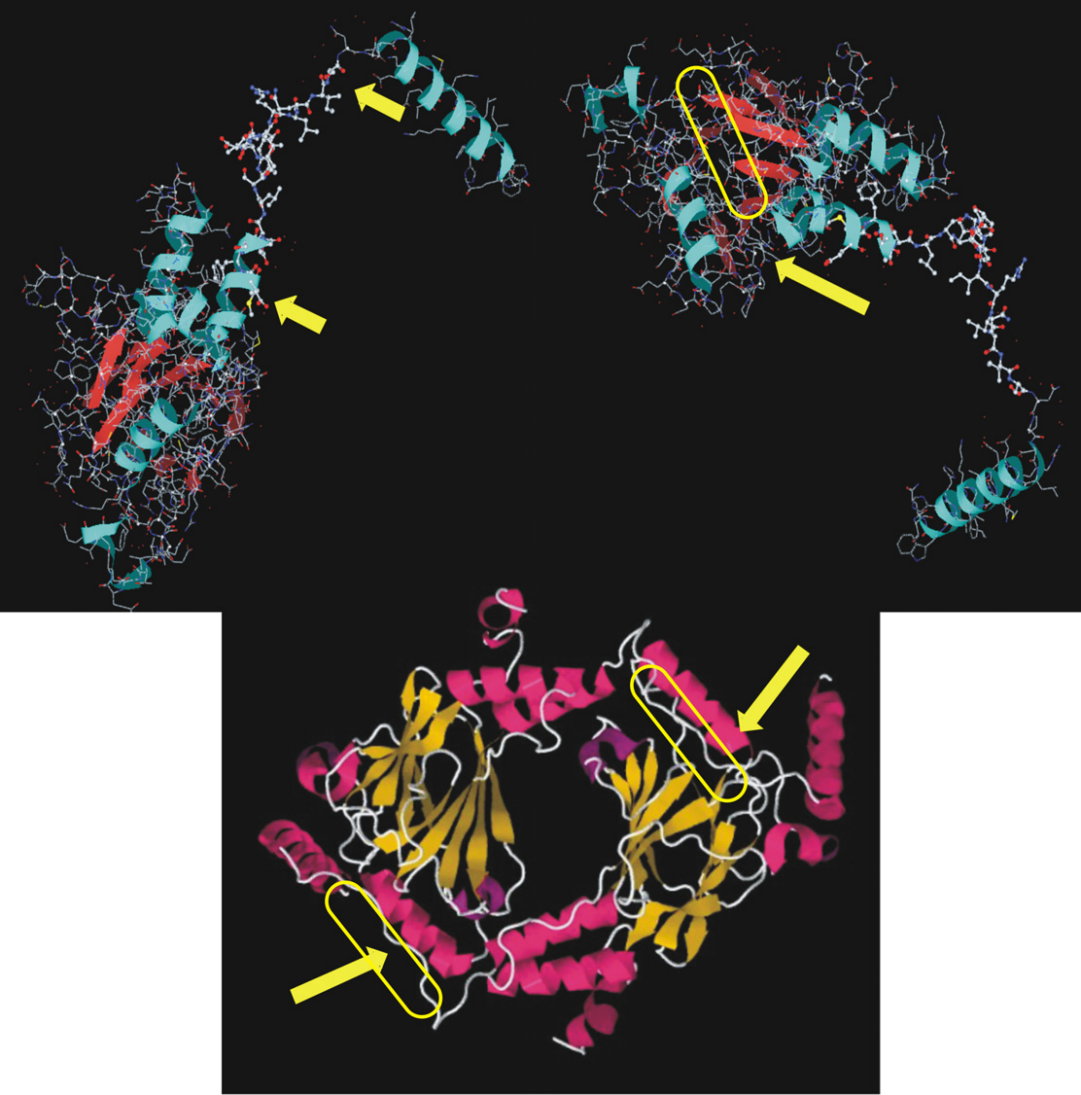
IRF5 3D Protein Structures. Asymmetric monomeric units of 3DSH (http://www.rcsb.org/pdb/explore/jmol.do?structureId=3DSH&bionumber=1) are shown in ball and stick format in different orientations using software from Medit SA (Palaiseau, France) (upper two figures). The upper left figure shows the region (between the two yellow arrows, amino acids (aa) 425–436) in IRF5 that is phosphorylated by a number of kinases. The upper right figure shows a yellow oval where IRF5’s phosphorylated tail domain is supposed to bind. The lower panel shows the homodimeric functional complex or biological assembly 1 unit in string and ribbon format using Jmol software from RSCB. The lower homodimeric figure of 3DSH is oriented to bring the cleft or valley to the left of the Helix 2 (aa303-312) into position for easier viewing. This is where the phosphorylated tail domain of IRF5 after serine phosphorylation is supposed to bind to form a homodimer. On the basis of the information in these 3D structures and reports of phosphorylation of IRF5 IRF5S (Ac-ELSWSADSIRLQISNPD-NH2) and IRF5D (Ac-ELDWDADDIRLQIDNPD-NH2) were designed.

In this section we explain how we designed IRF5 based on the 3D structure (3DSH) and mechanism of activation for IRF5 reported by Chen et al. in 2007 [18]. The region (between the 2 yellow arrows, amino acids (aa 425–436, Fig 1, upper left) in IRF5 is phosphorylated by a number of kinases [18,19]. The location of IRF5’s phosphorylation and binding domain is shown in Fig 1 (upper right panel). Using software from Medit SA (Palaiseau, France) structural features of the binding tail and cleft were examined. Using software from Research Collaboratory for Structural Bioinformatics (RSCB), the dimeric structure of 3DSH were rotated to better view how the tail docks in the cleft to the left of helix 2 (aa 303–312). Here is where the phosphorylated tail of IRF5 (activated state) is proposed to bind to form a functional homodimer complex. The design of the decoy peptide is as follows. The original sequence of the dimerization domain is ELSWSADSIRLQISNPD. This sequence was synthesized using standard FMOC-based solid phase techniques, end-capped with acetate and amide, and named IRF5S. To mimic phosphorylation aspartate (D) was substituted for serine (S) as was done previously to modulate eNOS activity [18,23]. The “phosphorylated” decoy peptide (ELDWDADDIRLQIDNPD) was synthesized, end-capped and named IRF5D.

### IRF5D binds IRF5

**Fig 2:**
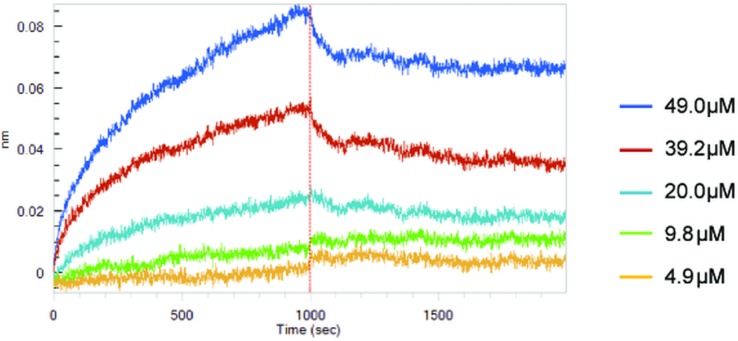
Senograms of IRF5D for IRF5. This figure shows binding curves of increasing concentrations of IRF5D for recombinant IRF5 determined by biolayer interferometry. Analysis of the curves using Octet Data Analysis software (v.7.1) showed that IRF5D binds to IRF5 with a Kd of 3.72 ± 0.74x10^-6^ M (mean±SD, n = 3).

Association studies indicated that IRF5D binds to recombinant IRF5 with a Kd of 3.72 ± 0.74x10^-6^ M (mean±SD, n = 3, Fig 2). In contrast, IRF5S was observed to bind to both naked biosensors and recombinant IRF5-loaded biosensors equally well (data not shown). What this means is that IRF5S does not bind to IRF5 with any appreciable affinity and that substituting aspartate (D) for serine (S) in the binding domain of IRF5S effectively enhances IRF5D association with IRF5 mimicking phosphorylation by substituting D for S.

### Effects of IRF5D on EC Proliferation and Apoptosis

**Fig 3:**
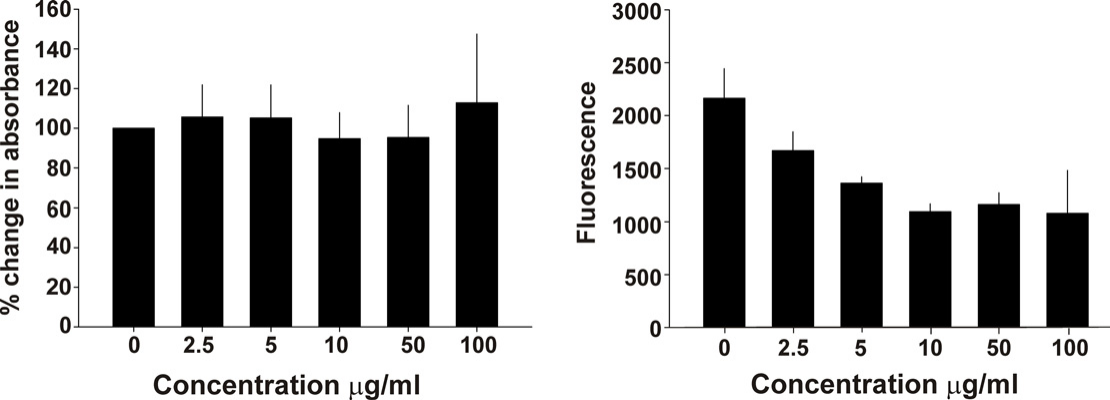
Effects of IRF5D on EC Proliferation and Apoptosis. These bar graphs show that increasing concentrations of IRF5D have no influence on EC proliferation (left, A) or apoptosis (right, B) as determined from the ability of the cultured EC to bioreduce the tetrazolium indicator dye or induce an increase in caspase activity as determined by MTS and caspase activity assays, respectively after incubation with IRF5D (0–100 μg/mL for 72 hours).

Increasing concentrations of IRF5D had little, if any, effect on EC proliferation based on the lack of change in MTS absorbance (Fig 3A). Likewise IRF5D treatments did not increase caspase activity in the EC cultures (Fig 3B). These data indicate that IRF5D does not impair EC proliferation or increase EC apoptosis.

### IRF5D Decreases Expression of IRF5 and ICAM-1 in Hearts of Tight Skin Mice

**Fig 4:**
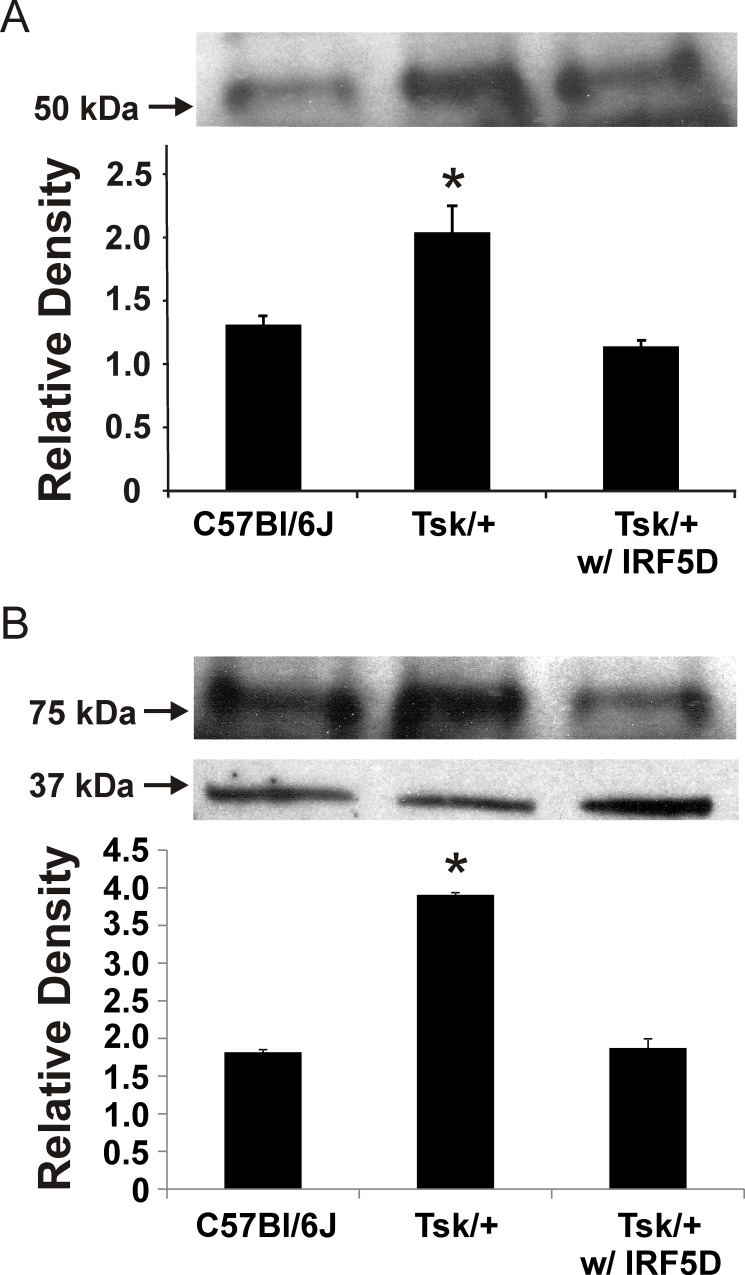
Effects of IRF5D on Myocardial ICAM-1 and IRF5 Expression. This figure shows western blots from heart homogenates from C57BL/6J mice as control and Tsk/+ mice treated with and without IRF5D (1 mg/kg/d) as described in Methods for ICAM-1 (top) and IRF5 (middle) relative to β-actin (bottom), a loading control. IRF5D treatments reduced ICAM-1 and IRF5 expression in the hearts of Tsk/+ mice compared to expression levels in hearts from Tsk/+ mice treated with PBS (p<0.05, n = 3).

Tsk/+ mice were used in the present report as an established murine model of myocardial inflammation and fibrosis. IRF5 and ICAM-1 expression in Tsk/+ hearts were increased nearly two-fold compared with expression levels in C57Bl/6J hearts (Fig 4). IRF5D treatments reduced IRF5 and ICAM-1 expression in Tsk/+ hearts (Fig 4). These data demonstrate that IRF5D effectively decreases myocardial inflammation in Tsk/+ mice.

### IRF5D Decreases Myocardial Monocyte/Macrophage and Neutrophil Counts

**Fig 5:**
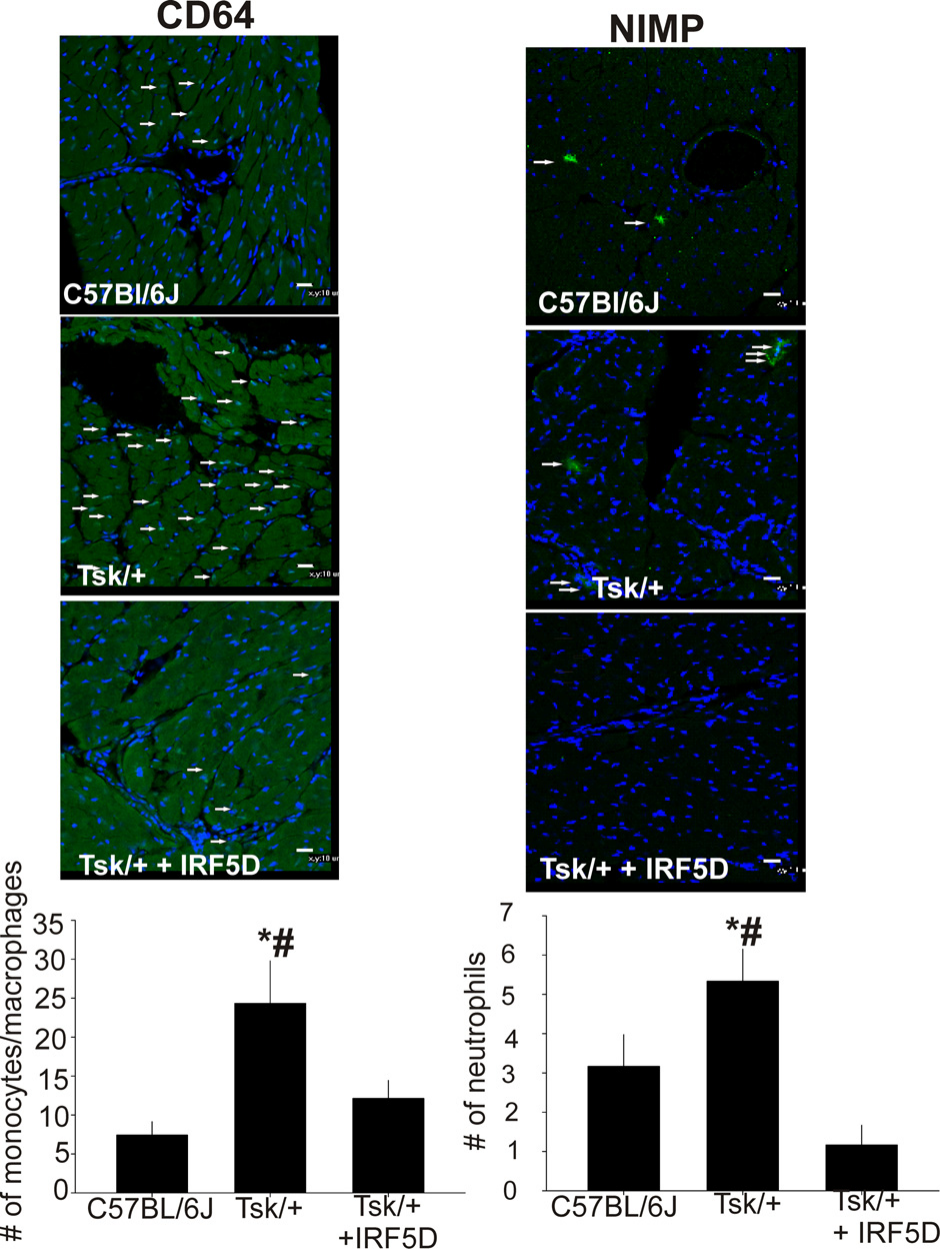
Effects of IRF5D on CD64 and NIMP expression. This figure shows immunohistochemistry of sections of the myocardium from C57BL/6J mice and the Tsk/+ mice with and without IRF5D treatment. The number of monocytes/macrophages and neutrophils determined by CD64 and NIMP fluorescence intensity is increased in Tsk/+ myocardium and IRF5D treatment reduces the number of monocytes/macrophages and neutrophils. (* = p<0.05, C57BL/6J vs. Tsk/+; # = p<0.025, Tsk/+ vs. Tsk/+ + IRF5D; n = 3, 10 images per antibody).

One of the hallmark features of inflammation in Tsk/+ mice is increased leukocyte infiltration in the myocardium. Fig 5 shows that the number of monocyte/macrophages and neutrophils is increased in the hearts of Tsk/+ mice compared with control mice. IRF5D treatments reduced the number of inflammatory cells in the hearts of Tsk/+ mice.

### IRF5D Improves EC Vasodilatation in Tight Skin Mice

**Fig 6:**
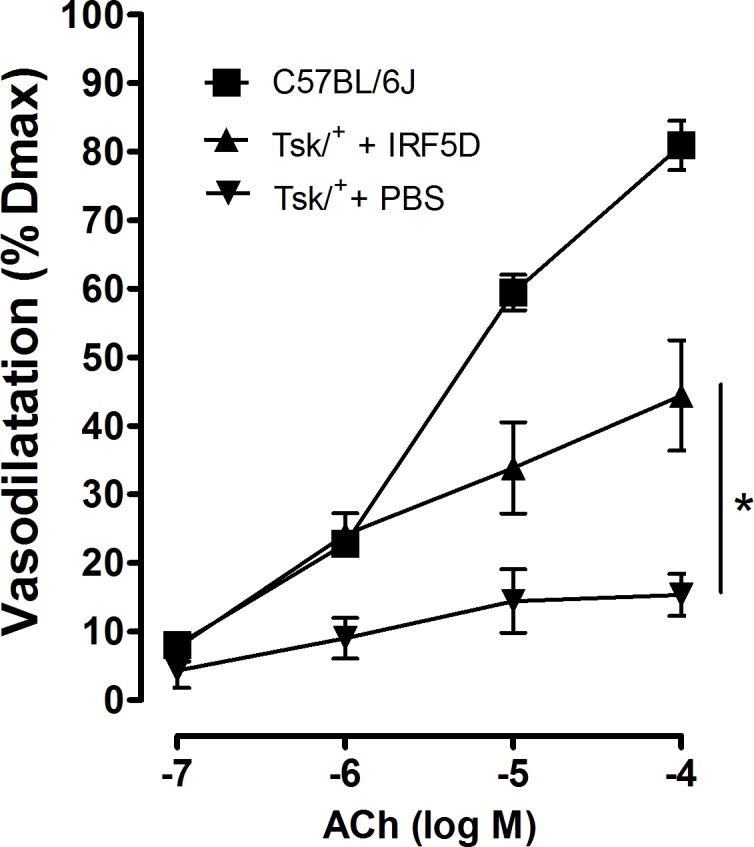
Effects of IRF5D on Vasodilatation. This line figure show the vasodilatation response curves for pressurized *facialis* arteries isolated from C57BL/6J control mice and Tsk/+ mice treated with IRF5D (1 mg/kg/d, 21 d) and Tsk/+ mice treated with PBS as described in Methods. The line graphs show that IRF5D treatments improved acetylcholine (ACh)–induced vasodilatation of *facialis* arteries in Tsk/+ mice compared to Tsk/+ mice treated with PBS (p<0.05, n = 6).

EC-dependent vasodilatation in *facialis* arteries isolated from Tsk/+ mice was impaired (~15%) compared with arteries isolated from C57Cl/6J mice (~85%, p<0.05, Fig 6). IRF5D treatments improved EC-dependent vasodilatation to ~45%, which is ~83% of vasodilatation observed in arteries isolated from C57BL/6J mice (Fig 6). These data indicate IRF5D improves EC-dependent vasodilatation in Tsk/+ mice.

### Effects of IRF5D on IRF5 Nuclear Translocation

**Fig 7:**
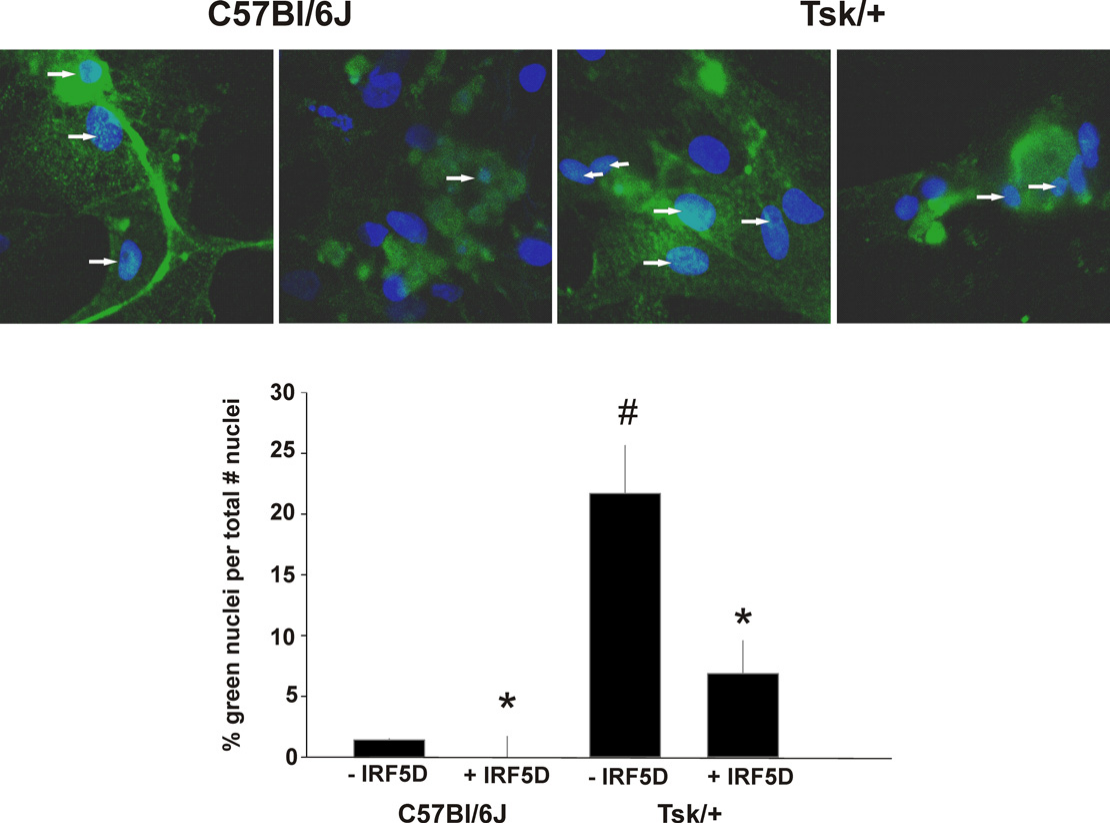
Effects of IRF5D on IRF5 Nuclear Translocation in Cultured Myocytes. The fluorescent images and bar graphs in this figure show the effects of IRF5D treatments on the number of IRF5 positive nuclei in myocytes cultured on C57Bl/6J (control) and Tsk/+ cardiac matrix. Tsk/+ cardiac matrix induces phenotypic changes in cultured myocytes (impaired spontaneous beating, and increased fibrosis that are consistent with injury [24]) that are reversed by IRF5D treatments (data not shown). Distribution of IRF5 in the cultured myocytes was determined as described in Methods. When IRF5 is in the nucleus the fluorescence of Alexa 488 combines with the fluorescence of the nuclear stain, DAPI, to form a pale blue color. The fluorescent images show greater numbers of IRF5 positive nuclei in myocytes cultured on Tsk/+ cardiac matrix than on C57BL/6J cardiac matrix and that treating the cultures with IRF5D (50 μg/mL, 24 h) reduced the number of IRF5 positive nuclei in myocytes cultured not only on Tsk/+ cardiac matrix but also C57Bl/6J matrix (p<0.05, n = 3).

Adding IRF5D directly to myocyte cultures maintained on Tsk/+ cardiac matrix decreased the number of IRF5+ nuclei from 23% to 9% (p<0.05, n = 3, Fig 7). Although the number of IRF5+ nuclei in myocyte cultures on C57Bl/6J cardiac matrix was much lower (~3%) than in myocytes on Tsk/+ cardiac matrix (~23%), adding IRF5D to myocyte cultures on control matrix reduced the number of IRF5+ nuclei even further to ~0.2% (Fig 7). These data indicate that IRF5D prevents IRF5 nuclear translocation in cultured myocytes.

## Discussion

The goal of this study was to design and develop a decoy peptide to inhibit IRF5 and determine its effects on myocardial inflammation and vascular EC function in Tsk/+ mice, a murine model of myocardial inflammation and fibrosis and impaired vasodilatation. Our studies showed that IRF5D binds to IRF5, does not affect caspase activity and proliferation, reduces myocardial inflammation and improves vascular function in Tsk/+ mice, reduces IRF5 translocation to the nucleus. The fact that IRF5D is derived from the binding tail of IRF5, mimics activated IRF5 and actually reduces myocardial inflammation and improves EC-dependent vasodilatation in Tsk/+ mice provides strong support for the idea that IRF5D targets IRF5. Our findings demonstrate that IRF5D is an important new tool for delineating IRF5-dependent mechanisms in myocardial inflammation and fibrosis. Accordingly, IRF5D may be useful for investigating the role of IRF5 in inflammatory and fibrotic heart disease in a variety of autoimmune diseases.[12]

In the present study we showed that Tsk/+ cardiac matrix increases expression of inflammatory cell markers and that IRF5D treatment reduces their expression. Hearts of IRF5D-treated Tsk/+ mice had notably less ICAM-1 expression than the hearts of non-treated Tsk/+ mice, which is important because reductions in ICAM-1 are considered evidence of decreased vascular inflammation [25,26]. The fact that IRF5D reduces myocardial inflammation and increases vasodilatation begins to explain why 4F was so effective at decreasing inflammation and improving vasodilatation in Tsk/+ mice [12].

Genome-wide association studies (GWAS) are used to identify genes involved in disease. Once associations have been identified, they can be used to develop strategies to detect, treat and even prevent disease. Studies here grew out of GWAS showing that *IRF5* was associated with SSc [5–7] and other autoimmune diseases that increase heart disease and our knowledge that 4F inhibited IRF5 in Tsk/+ mice [12]. IRF5D made it possible for us to determine the extent to which IRF5 was involved in myocardial inflammation simply by treating the mice and observing changes in IRF5 activation and ICAM-1 in Tsk/+ hearts.

Although studies using knockout mice are considered by many to yield data that are definitive, knockout data does not provide any information as to whether a given gene product is druggable. In contrast, if a decoy or blocking peptide inhibits the biological activity of that target protein, alters the course of disease and/or restores vascular function because of its interaction with the target protein, this is strong evidence that the gene product is directly involved in disease and more importantly that the gene product is druggable, which has profound translational implications.

The fact that IRF5D binds IRF5, decreases IRF5 expression and activation and decreases myocardial inflammation provides strong proof that not only is IRF5 druggable but that IRF5D is a highly effective therapeutic agent for targeting mechanisms that increase myocardial inflammation and fibrosis to protect the heart. Accordingly, IRF5D provides new opportunities for investigators to determine if and the extent to which inhibiting IRF5 is an effective for treating myocardial and vascular disease in autoimmune disease.

In conclusion our findings show that IRF5 plays an important role in the mechanisms driving myocardial inflammation and fibrosis. Accordingly, inhibiting IRF5 may be an effective therapeutic strategy for treating myocardial inflammation and fibrosis in humans with autoimmune disease [27].
